# Zebra Risk Perception in a Landscape of Fear

**DOI:** 10.1002/ece3.71275

**Published:** 2025-05-14

**Authors:** Yuchen Chen, Daniel T. Blumstein, Diana M. Boyle, Natasha Bartolotta, Jessica Brown, Bernard Kissui, Matthias Waltert, Christian Kiffner

**Affiliations:** ^1^ Department of Ecology and Evolutionary Biology University of California Los Angeles Los Angeles California USA; ^2^ Department of Conservation Biology Georg‐August Universität Göttingen Göttingen Germany; ^3^ Cornell University Ithaca New York USA; ^4^ University of St. Thomas St. Paul Minnesota USA; ^5^ Center for Wildlife Management Studies The School for Field Studies Karatu Tanzania; ^6^ Research Area 2 ‘Land Use and Governance’ Leibniz Centre for Agricultural Landscape Research (ZALF) Müncheberg Germany; ^7^ Thaer‐Institute of Agricultural and Horticultural Sciences Humboldt‐Universität zu Berlin Berlin Germany

**Keywords:** antipredator response, flight initiation distance, group size, landscape of fear, predation risk, vigilance

## Abstract

Animals' assessments of predation risk are influenced by a variety of external and internal factors, including predator space use. However, it remains unclear what variables mediate prey species behavior within a landscape where predation risk is heterogeneous. To address this, we employed three assays to examine zebra ( *Equus quagga* ) responses to varying predation risk in a multiple‐use area of northern Tanzania: (1) quantifying head‐up posture as a proxy for vigilance through direct behavioral observation in areas of high and low likelihood of lion ( *Panthera leo* ) presence, (2) quantifying head‐up posture as a proxy for vigilance when exposed to a lion roar playback, and (3) measuring flight initiation distances (FIDs) when approached by a person. Using generalized linear (mixed) models, we tested how lion space use and habitat type (as proxies for predation risk), normalized difference vegetation index (NDVI, as proxy for primary productivity), time of the day, and zebra‐related variables (sex‐age category, zebra herd size, group size including heterospecifics, and location within the herd) influenced vigilance and flight responses. We found that (1) neither vigilance nor FID were markedly influenced by estimated lion space use, habitat type, and NDVI; (2) vigilance decreased with group size, was lower for zebras positioned centrally in the herd, and during midday; (3) FID increased with a greater number of associated heterospecifics; and (4) zebras increased vigilance when exposed to lion roar playbacks, irrespective of lion space use. These findings suggest that zebra vigilance and flight behavior are not necessarily mediated by spatial variation in apparent predation risk but instead reflect a strategy of maintaining a consistent monitoring of possible threats across the landscape. Rather than relying on spatial clues alone, zebras primarily mitigate predation risk by increasing group size and associating with other species.

## Graphical abstract

We examined how zebras ( *Equus quagga* ) in northern Tanzania respond to predation risk from lions ( *Panthera leo* ) and humans using behavioral observations, lion roar playbacks, and flight initiation distance (FID) assays. Vigilance decreased with group size and central herd positioning, while FID increased with heterospecific associations, but neither was strongly influenced by lion space use, habitat, or NDVI. These findings suggest that zebras mitigate predation risk through grouping and consistent threat monitoring rather than relying solely on spatial risk cues.

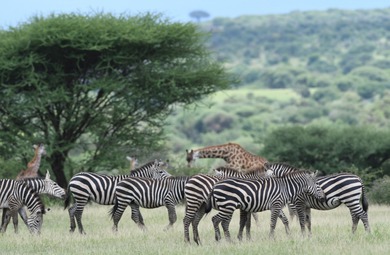

## 1 Introduction

Predators can shape the behavior, distribution, and population dynamics of prey species (Brown et al. 1999). Facing predation pressure, animals have evolved a variety of antipredator responses, including seeking refuge (Lammers et al. 2009), adjusting group size (Tambling et al. 2012), and increasing vigilance (Hunter and Skinner 1998). Decades of research across various taxa have identified a suite of factors that influence risk assessment, including the predator type, prey traits, environmental conditions, and the roles of experience and learning (Stankowich and Blumstein 2005).

Predation involves several stages including encounter, detection, interaction, attack, and capture (Lima and Dill 1990). Correspondingly, prey species have evolved multiple strategies to evaluate their risks at each stage. These assessments include evaluating the likelihood of being noticed by predators, the chance that being noticed will lead to an attack, the probability that an attack will result in a kill, and, ultimately, the chance of being the individual prey killed (Creel 2018). Therefore, we can also study antipredator responses in different stages, such as before, during, and after an encounter.

Evaluating and reacting to predation risk is costly. Prey may increase their physiological stress levels (Boonstra et al. 1998) as well as have reduced foraging efficiency and restricted food choices (Sinclair and Arcese 1995). These costs have demographic consequences and may be associated with reduced reproduction and population growth rates (Creel et al. 2014). Therefore, it is important to understand under what circumstances, to what degree, and how animals perceive and react to predation risks.

**FIGURE 1:**
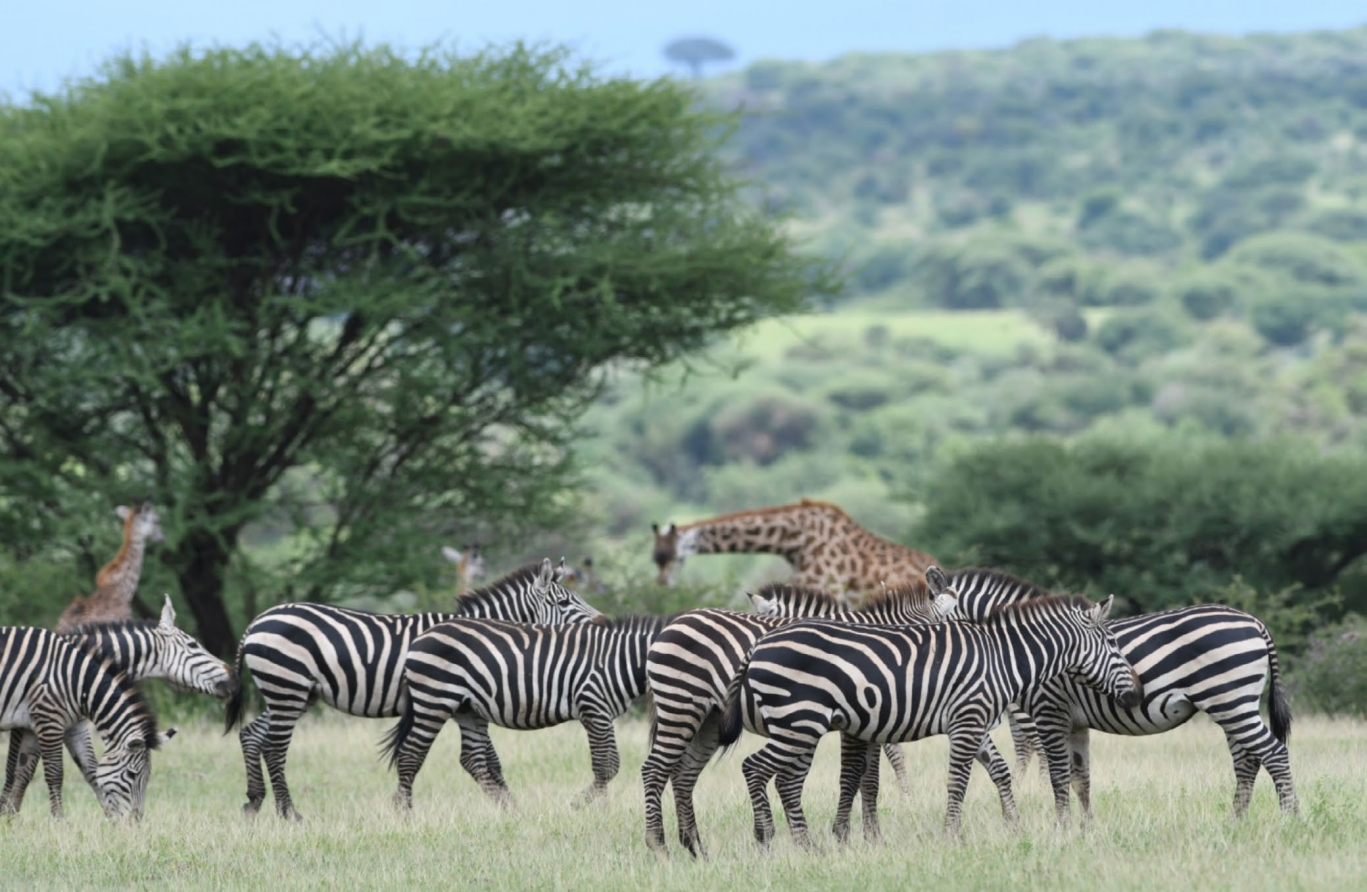
Zebra ( *Equus quagga* ), forming a mixed species group with giraffe ( *Giraffa tippelskirchi* ) in Manyara Ranch, northern Tanzania (Photo: Christian Kiffner).

Ungulates, such as the plains zebra ( *Equus quagga* , Figure 1), are preyed upon by a variety of large carnivores and fall within the preferred prey weight range of African lions ( *Panthera leo* ) (Hayward and Kerley 2005). Previous studies have examined various antipredator behaviors in African ungulate species (Palmer and Packer 2021; Creel et al. 2014; Gehr et al. 2018). For example, zebras are more likely to flee immediately after an encounter in bushy areas and to select open grasslands during their flight (Courbin et al. 2016). Similarly, blue wildebeest ( *Connochaetes taurinus* ) and zebras ( *Equus quagga* ) increased group sizes when lions were present (Thaker et al. 2010), and giraffe ( *Giraffa camelopardalis* ), African buffalo ( *Syncerus caffer* ), and greater kudu ( *Tragelaphus strepsiceros* ) avoided waterholes when lions were in the vicinity (Valeix et al. 2009).

The landscape of fear theory outlines how prey species' behaviors and distributions are shaped by perceived spatial variation in predation risks (Laundré et al. 2001). Many studies have explored how the spatial distribution of predators influences prey behavior (e.g., Parker et al. 2022). For example, snow geese exhibit more intense nest defense behaviors in regions frequently visited by foxes (Clermont et al. 2021). Conversely, elk ( *Cervus elaphus* ) were found to be less vigilant in areas with high exposure to wolves compared to those with infrequent exposure (Creel et al. 2008).

With increased human presence in wildlife habitats, the role of anthropogenic threats needs to be incorporated when characterizing the landscape of fear (Ciuti et al. 2012; Moleón and Sánchez‐Zapata 2023). Several studies suggest that prey species evaluate the risk posed by humans based on the likelihood of their natural predators being present in a given area. Generally, the flight initiation distances (FID) in response to an approaching human tend to be greater in locations where natural predators are present than in predator‐free areas, as observed in birds (St Clair et al. 2010) and reptiles (Berger et al. 2007).

Several predators, including lions, are territorial and preferentially hunt in specific areas, creating areas of acute mortality risk for their prey (Hopcraft et al. 2005). However, little is known about how prey species assess risks from both predators and humans in landscapes with varying levels of predation risk, and what factors mediate this assessment, such as habitat type, food availability, time of day, and grouping patterns. Narrowing this knowledge gap is crucial for advancing our understanding of the landscape of fear, animal risk assessment, and the evolution of antipredator responses.

To address this, we examined how zebras perceive and react to predation risks from lions and humans in a multi‐use area of northern Tanzania. Lions are the primary predators of zebras in our study site, and illegal hunting is a threat both inside and outside protected areas (Kiffner et al. 2015). To analyze zebra risk assessment, we used three complementary approaches.

First, we estimated zebra vigilance using instantaneous scan sampling, defining vigilance as actively monitoring for potential threats, indicated by a raised head with eyes above shoulder height.

Second, we quantified the response of zebras to playbacks of lion roars, again using instantaneous scan sampling to quantify raised head posture. Most predators do not vocalize when hunting, yet a variety of prey species can discriminate and react to the sound of predators (Hettena et al. 2014). Playback experiments have been shown to be effective in simulating predator presence and assessing antipredator responses across various taxa, including ungulates (Favreau et al. 2013; Zanette et al. 2023), primates (Adams and Kitchen 2020), and birds (Forsman and Mönkkönen 2001). Specifically, we predicted that the magnitude of the response to the lion roar playback would not be influenced by lion space use because a lion roar represents a direct, strong predation cue to which zebras should respond, regardless of their location.

Third, we quantified flight initiation distances (FID) of zebras to simulate anthropogenic threats. FID is defined as the distance at which animals start to flee from an approaching predatory threat, including humans (Ydenberg and Dill 1986), and is a common method for quantifying perceptions of predation risk (Stankowich and Blumstein 2005). However, the extent to which FID reflects generalizable risk perception beyond specific threats remains an open question (Allan et al. 2021). If fear is generalizable, FID in response to an approaching human should be greater in areas where encounters with natural predators are more likely. Conversely, if fear is context‐specific, FID may primarily reflect responses to (or prior experience with) human disturbances.

We considered several factors that could mediate zebra risk assessment and hypothesized that demographic characteristics contribute to variation in risk perception. Specifically, we expected females with offspring to be more attuned to potential threats (Lima and Dill 1990). Additionally, we predicted that zebras in larger aggregations (whether in zebra‐only or in multi‐species groups) and zebra individuals positioned at the center of a herd would reduce their individual investment in predation risk assessment (Beauchamp 2015). Because many ungulates must balance energy intake with predator avoidance (Kie 1999), we included primary productivity (NDVI) as a proxy for food availability and expected that zebras in areas with high NDVI would invest less in risk perception. Since zebras in bushier areas have been shown to perceive greater risk (Chen et al. 2021), we included habitat types to estimate small‐scale risks. Finally, we hypothesized that zebra risk perception would be highest in the morning (when lions are presumably more active) compared to midday (Dröge et al. 2017; Kittle et al. 2022).

## 2 Methods

### 2.1 Study Area

**FIGURE 2:**
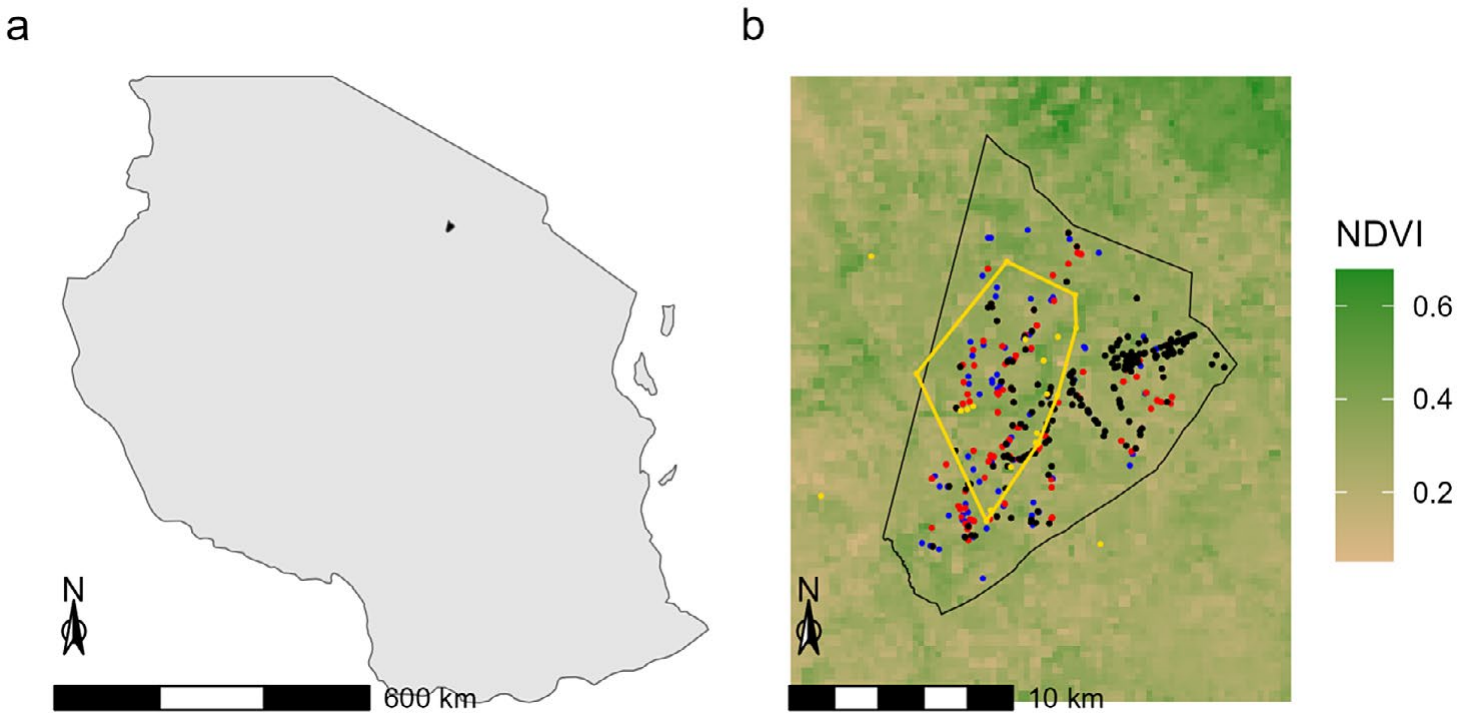
Map of the study area. (a) Shows the location of the study area in Tanzania. (b) Illustrates the location of the zebra observations (black = behavior scans; red = behavior scans with playbacks; blue = flight initiation distance experiments) in relation to the lion home range (gold dots = location of lion observations, gold polygon = 85% minimum convex polygon) and the average NDVI scores during the study period.

We studied zebras in Manyara Ranch (MR), situated within the Tarangire ecosystem of northern Tanzania (Figure 2). This 183 km^2^ multiple‐use area, formerly managed as a cattle ranch by different entities from 1956 to 2001, has been managed to support both wildlife conservation and pastoralism since 2001 (Kiffner et al. 2020a). During our study period, the ranch management owned 800 Boran cattle ( *Bos indicus* ) and 400 Somali sheep ( *Ovis aries* ) who grazed the area. In addition, adjacent Maasai pastoralists are allowed to graze their livestock here during the dry season (Warwick et al. 2016). Despite patrols by rangers enforcing anti‐poaching and grazing regulations, illegal hunting for local wild game markets occasionally occurs in MR (Kiffner et al. 2014).

The area is characterized by a semi‐arid climate with a bimodal rainfall pattern: precipitation mostly occurs during the long rains (February to May) and the short rains (November to December) and ranges from 415 to 844 mm annually (Beattie et al. 2020; Prins and Loth 1988). The vegetation is dominated by *Acacia* (*Vachellia*)‐*Commiphora* savanna consisting of a patchwork of open grasslands and areas with bush and tree cover. Several man‐made dams and the Makuyuni River, which bisects the area, provide surface water year‐round. During the long rains (i.e., when we conducted fieldwork for this study), surface water is widely available across the landscape.

The area plays an essential role in the annual migration of zebra and wildebeest populations within the Tarangire ecosystem (Lohay et al. 2022; Riggio et al. 2022), serving as a crucial steppingstone during their annual round‐trip between their core dry season range in adjacent Tarangire National Park and their wet season calving grounds in the northern plains near Lake Natron (Morrison and Bolger 2012). The availability of grass and surface water attracts zebras and wildebeests throughout the year, with densities peaking at the onset of the dry and wet seasons due to migratory movements (Kiffner et al. 2020a). The species composition and densities of most herbivorous mammals in MR are comparable to those in the adjacent Tarangire National Park (Kiffner et al. 2016; Kiffner et al. 2020b).

For our research, MR offered an ideal environment for several reasons. First, the area's high zebra density facilitated data acquisition; during our study period (February to April 2015), we estimated a zebra density of 10.4 (95% CI: 6.4; 16.8) individuals per km^2^ (Kiffner et al. 2020a). Second, in MR, lions are the principal natural predator of zebra during the daytime, which reduced the possibility of confounding effects caused by other predators. An extensive camera‐trap study conducted a few months prior to this study (Beattie et al. 2020) confirmed the presence of spotted ( *Crocuta crocuta* ) and striped hyenas (*Hyena hyena*) but indicated that these species were strictly nocturnal. Leopards ( *Panthera pardus* ), cheetahs ( *Acinonyx jubatus* ), and African wild dogs ( *Lycaon pictus* ), though very infrequently sighted by ranch personnel, were neither detected during our camera trap survey nor reported throughout the duration of our study.

### 2.2 Data Collection Overview

Fieldwork for this study was carried out with permission from the Tanzania Wildlife Research Institute (TAWIRI) and the Tanzania Commission for Science and Technology (COSTECH) (permit #: 2014‐324‐ER‐2013‐191) and explicit permission from the management of Manyara Ranch.

We conducted this study during the long rains, from 19 February through 20 April 2015. To locate zebras, we navigated the minor tracks of MR, ensuring we altered routes daily to avoid pseudoreplication (i.e., unknowingly sampling the same individual zebra more than once). Although we minimized repeat sampling by alternating routes and covering different areas of the study area and given the high density of zebra in our study site (10.4 (95% CI: 6.4–16.8) zebra/km^2^), equating to approximately 1900 zebras (Kiffner et al. 2020a), we cannot entirely exclude the possibility of resampling the same individual. Similarly, in the playback experiments, some zebras may have been exposed to the stimuli more than once. We mitigated this by spacing playback experiments several kilometers apart on a given day. Upon encountering a zebra herd, which we defined as individuals located within 50 m of one another (Kiffner et al. 2014), we stopped the vehicle at a distance that allowed us to observe the zebras without causing them undue disturbance and turned off the engine. To investigate how zebra risk perception was influenced by a suite of relevant conditions, we compiled three datasets.

### 2.3 Quantifying Vigilance

Observing from open‐top Land Cruisers at a distance of ca. 100 m (101.2 m (standard deviation [SD] = 8.6 m)), we allowed a few minutes for the zebras to minimize immediate disturbance. As zebras in our study area are not strongly habituated and often walk or run away from vehicles (Kiffner et al. 2014), we maintained this distance to minimize observer influence while still allowing for accurate behavioral observations.

We then used instantaneous sampling to monitor the behavior of focal individuals, recording their behaviors every ten seconds over a 2‐min period (average number of scans per zebra: 11.9; median: 12; range: 7–13). We categorized behaviors into four categories: vigilant (i.e., head raised with eyes above shoulder height while standing still), feeding (i.e., head lowered below shoulder height and grazing), locomotion (i.e., animal moving), and other behaviors (e.g., nuzzling, mating, nursing). These observations were facilitated by the use of 10 × 42 binoculars and were conducted by the same, trained observers. We selected around 6 individual zebras from each herd (median: 6; mean: 6.22; range: 1–18) in a quasi‐random manner, ensuring that we included individuals from both the interior and the periphery of the herd. Lacking an aerial view of the herd, we adopted a basic classification for the positioning of individuals within the herd. This was based on an observational assessment of each zebra's likelihood of being the first point of contact for a predator approaching from any direction. We classified positions into three categories: (1) on the herd's perimeter without any buffer, (2) in the herd's core with a zebra buffer, or (3) in the core with a heterospecific buffer. We sampled a total of 1257 zebras in 202 herds.

### 2.4 Response to the Lion Playback

The data recording in this experiment followed the same protocol as outlined in the previous section, sampling around 5 individual zebras from each herd (median: 5; mean: 4.97; range: 1–10) in a quasi‐random manner, ensuring that we included individuals from both the interior and the periphery of the herd and recording their behaviors every 10 s over a 2‐min period (average number of scans per zebra: 11.6; median: 12; range: 3–12).

We randomly subjected zebra herds to one of the following audio stimuli: the playback of a lion roaring, the call of a fish eagle (*Halieaeetus vocifer*), and silence. For these experiments, we broadcast the sounds from a distance of 101.2 m (SD = 9.6 m), continuously looping the sound for the entire 2 min duration at full volume using a FOXPRO Firestorm digital wildlife caller. Prior to conducting the fieldwork, we normalized the audio levels of the two sound files using the Audacity 2.1.2 software. Using the dB meter app (Splend apps), we measured the peak amplitude at a 1 m distance, the fish eagle playback was 97 dB, and the lion sound 103 dB. For this experiment, we sampled all individuals simultaneously within a herd and recorded the behavior of 631 zebras in 127 herds.

### 2.5 Flight Initiation Distance

Prior to each FID experiment, the research team and a ranger scanned the area to ensure that no potentially dangerous animals (e.g., elephant, buffalo, and lion) would compromise safety. The same person (Natasha Bartolotta), consistently dressed in a white T‐shirt and khaki pants, approached a zebra herd at a steady pace, starting from an average distance of 200.1 m (SD = 6.3 m). The observer measured the distance from the zebra herd where they started their approach (the starting distance), the distance where zebras were initially responding to the approaching human (first response; typically, zebras slowly moving in response to the approaching human), and the distance from where zebras began to run away from the approaching human (the flight initiation distance, FID). We defined a ‘flee’ response as running away not just simply walking. Because zebras typically reacted collectively (i.e., if one zebra started running, the others ran as well), we measured a single FID for each of the 90 herds that we approached. We defined the FID as the onset distance of a running event of a herd. All distances were measured using a laser range finder (Leupold RX‐1000ri).

### 2.6 Explanatory Variables

For every zebra sampled, we recorded its sex‐age class, limiting our sampling to yearlings (which were not sexed) and both adult females and males (thus we did not include foals in the behavioral scans). For the vigilance and playback assays, we also recorded the positioning of each zebra within the herd (for details see *quantifying vigilance*). To map the experiments, we recorded the GPS coordinates of the vehicle; we did not attempt to further pinpoint the exact location of the zebra herd. To describe the habitat around each zebra herd, we assigned one of four habitat classes based on vegetation physiognomy (grassland, open bushland, bushland, and shrubland). We conducted vigilance assays during morning (defined as 07:40–10:30), midday (10:31–13:30), afternoon (13:31–16:30), and evening (16:31–19:00), and conducted playback and FID experiments during the morning, midday, and afternoon.

The Tarangire Lion Project monitored lions on MR from 2010 to 2015, enabling us to estimate their space use. In light of the limited sample size (*n* = 23 data points on lion occurrence from 2010 to 2015, possibly from multiple prides), we used the minimum convex polygon method with 85% isopleths to estimate the lion space use (LSU). Using the “sf” package in the software R (v1.0.15; Pebesma and Bivand 2023) and geographical coordinates, we categorized each individual zebra (vigilance and playback datasets) or zebra herd (FID dataset) as either within or outside of the LSU. In the vigilance dataset, 269 zebras were within and 988 were outside the LSU (*n* = 1257). In the playback dataset, 317 zebras were within and 314 were outside the LSU. In the FID dataset, 39 zebra herds were within and 51 were outside the LSU. While we cannot exclude the possibility that resident or dispersing lions used areas outside of the LSU, all subsequent opportunistic lion observations (*n* = 3) made by author CK between 2015 and 2019 occurred within the delineated LSU.

As a measure for primary productivity, we obtained time‐matched values of the normalized difference vegetation index (NDVI) using Google Earth engine. Using the NDVI layer from the MODIS Terra vegetation index product (MODI3Q1 V6) and implementing a 200 m buffer around the observation location to account for discrepancies between the actual zebra location and the GPS coordinates recorded, we calculated the average NDVI score for each intersecting cell, weighted by area. We selected NDVI scores from the day that was closest to each field experiment. The difference between the observation date and the satellite imagery date ranged from 0 to 16 days. In line with previous research on this dataset, we binned NDVI scores in three equal‐sized classes (small, medium and large), using 0.33 and 0.67 percentiles (Kiffner et al. 2022). In the vigilance dataset, 408 observations were categorized as large NDVI, 431 as medium, and 418 as small. In the playback dataset, 206 observations were categorized as large NDVI, 217 as medium, and 208 as small. In the FID dataset, each NDVI class includes 30 experiments.

### 2.7 Data Analysis

All statistical analyses were conducted using R software (v4.3.2; R Core Team 2023) using the “glmmTMB” package for model fitting (v1.1.8; Brooks et al. 2017). To visualize our data points, using the “ggplot2” package (v3.4.4; Wickham 2016), we plotted individual zebra observations from behavior scans and playback experiments and flight initiation distance (FID) trials onto a map (Figure 1). On the same map, we included MR and LSU as polygons, and NDVI scores as a gradient.

For both vigilance and playback datasets, the target variable was the number of vigilance scans recorded for each scanned zebra. For these datasets, we used a generalized linear mixed modeling approach using the counts of vigilance as the response and the total number of scans as an offset.

Initial analysis indicated signs of overdispersion and excess zeros in both the vigilance and the playback datasets. To choose a suitable model specification for each of the datasets, we conducted model selection. We specified models with either negative binomial error distributions type 1 or type 2, both with a log link function. In addition, to handle zero inflation, we fitted several models: a model that does not account for zero inflation, a simple zero‐inflated model with an intercept only, and two zero‐inflated models with predictor variables that were either strongly associated with vigilance (position within the herd) or key to our research question (location relative to the lion home range). We used the Akaike information criterion corrected for small sample sizes (AICc) as the metric for model selection, comparing the AICc values to select the most appropriate model structure.

We chose this approach to rigorously test our hypotheses regarding the factors influencing vigilance behavior, rather than to develop predictive models. While our model accounts for overdispersion and zero inflation, it does not explicitly constrain counts to their upper limit (i.e., the maximum of behavioral scans per zebra) and hence does not capture all distributional constraints in the data.

### 2.8 Antipredator Vigilance

For the vigilance dataset, model selection favored a model with negative binomial error distribution type 1, which accounted for excess zeros by including the position of the individual zebra within the herd (at the edge of a zebra group, inside a zebra group, and inside a heterospecific group) to differentiate between zero vigilance and counts of vigilance (Appendix A, Table A1). As fixed effects for the count part of the model, we included the following variables: time of the day (morning, midday, afternoon, and evening), demographic characteristics (sex‐age class of the observed zebra), position of the zebra (at the edge of a zebra group, inside a zebra group, and inside a heterospecific group), herd size of the zebras, total herd size (in mixed species group; this included all zebras plus any heterospecific mammals), and habitat type (grassland, open bushland, shrubland, and bushland), whether zebras were inside or outside the LSU, NDVI Class (small, medium, and large), and the presence of zebra foals (i.e., zebras born in the current rainy season). We included herd ID as a random effect to account for the non‐independence of observations of individual zebras within the same herd.

### 2.9 Response to Playback

Model selection suggested a model structure with negative binomial error distribution type 1 that accounts for excess zeros by including an intercept‐only model part to differentiate between zero vigilance and counts of vigilance (Appendix A, Table A2). As for the vigilance model, we included the same fixed effects including time of the day: morning, midday, and afternoon, whether zebras were inside or outside the LSU, the zebra's sex‐age class (male, female, and yearling), position of the zebra (at the edge of a group, inside a zebra group, and inside a heterospecific group), zebra herd size, total herd size of the mixed species group, habitat type (grassland, open bushland, shrubland, and bushland), NDVI Class (small, medium, and large), presence of foals, and the playback stimulus (lion, absent, and fish eagle). In addition, we added an interaction between playback stimulus and whether the experiment was conducted inside the LSU. We included herd ID as a random effect to account for non‐independence of observations within herds.

### 2.10 Flight Initiation Distance

We fitted a generalized linear model with Gaussian distribution to explain variation in flight initiation distances among zebra herds. We considered a number of fixed effects, including time of the day (morning, midday, and afternoon), habitat type (grassland, open bushland, shrubland, and bushland), zebra herd size, total herd size of the mixed species group, whether zebras were inside or outside the LSU, NDVI Class (small, medium, and large), whether there were zebra foals present, number of other species in the herd, and start distance (because this often explains a significant proportion of variation in FID, Blumstein et al. 2015).

### 2.11 Model Evaluation

We standardized continuous independent variables to facilitate direct comparison. Using the check_model function of the performance package (v0.10.9; Lüdecke et al. 2021), we evaluated models for vigilance with and without playbacks using six measures (posterior predictive check, overdispersion and zero‐inflation, homogeneity of variance, collinearity, normality of residuals, and normality of random effects). To evaluate the model for the FID, we computed five measures (posterior predictive check, linearity, homogeneity of variance, collinearity, and normality of residuals). In addition, we estimated conditional and marginal R^2^ using the performance function of the performance package (v0.10.9; Lüdecke et al. 2021).

## 3 Results

### 3.1 Antipredator Vigilance

The model explaining variation in head‐up posture fit the data well and appropriately handled overdispersion and zero‐inflation. The variance of residuals was homogeneous, collinearity was minimal, and both the residuals and random effects followed a normal distribution (See Appendix C, Figure C1). The model had a conditional *R* ^2^ value of 0.751, indicating both fixed and random effects together explained 75.1% of the observed variation. However, the marginal *R* ^2^ value was 0.154, suggesting that the fixed effects alone explained only 15.4% of the observed variance. In turn, this suggests that herd identity substantially influenced vigilance behavior.

**FIGURE 3:**
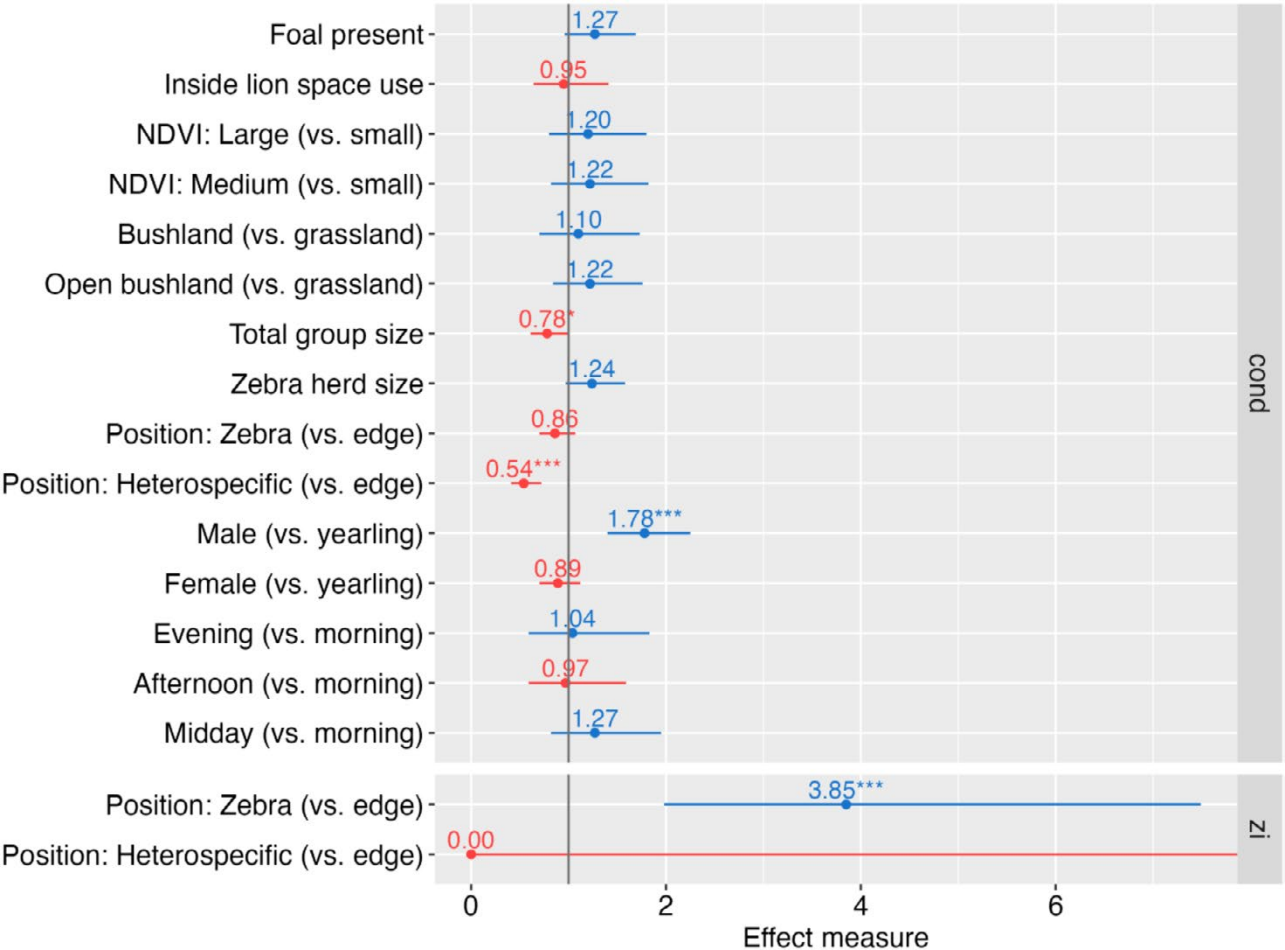
Effect measures of a generalized linear mixed model with negative binomial error distribution. The model consists of a zero‐inflation part (zi: Effect measures = odds ratios) which accounts for excess zeros, and a conditional part (cond: Effect measures = incidence rate ratios) which models the counts of vigilance scans. For the zero‐inflation sub‐model, we included the zebra's position within the herd as a predictor. The conditional (count) sub‐model assesses the impact of multiple environmental and zebra‐related variables on the proportion of vigilant scans of zebra. The proportion of vigilant scans is based on behavioral observations in Manyara Ranch, northern Tanzania.

Among the fixed effects, we found that male zebras exhibited significantly higher vigilance than yearlings (exp(β) = 1.78; 95% CI: 1.40–2.25, *p* < 0.001), while vigilance in female zebras was similar to that of yearlings (exp(β) = 0.89; 95% CI: 0.70–1.12, *p* = 0.322; see Figure 3 for effect measures and Appendix B, Table B1). The total group size (which includes the number of zebra and other prey species) was negatively correlated with the proportion of vigilant scans, whereby larger groups displayed lower vigilance (exp(β) = 0.78; 95% CI: 0.61–1.00, *p* = 0.047). Position within the herd influenced both the logistic regression and the conditional part of the model. In the conditional portion of the model, zebras with a heterospecific neighbor at the edge exhibited a significantly lower vigilance level (exp(β) = 0.54; 95% Cl: 0.41–0.72; *p* < 0.001) than zebras without a neighbor at the edge. Zebras buffered by a different zebra at the edge of the herd had intermediate vigilance levels (exp(β) = 0.86; 95% Cl: 0.70–1.07; *p* = 0.172). However, the logistic regression part of the model suggests that zebras located inside a zebra group had greater odds of being vigilant (exp(β) = 3.85; 95% Cl: 1.98–7.49; *p* < 0.001), whereas a heterospecific neighbor at the edge did not mediate the odds for vigilance (exp(β) = 0.00; 95% Cl: 0.00‐Inf; *p* = 0.996) compared to zebras located at the periphery of a herd. Other variables, including location relative to the LSU, time of the day, habitat type, NDVI class, zebra herd size, and presence of a foal, did not significantly influence the proportion of vigilance scans.

### 3.2 Response to Playback

The model explaining variation in head‐up posture during the playback experiments fit the data well and appropriately handled overdispersion and zero‐inflation. The variance of residuals was homogeneous, there was moderate collinearity, and the residuals and random effects followed a normal distribution (See Appendix C, Figure C2). The model had a conditional R^2^ of 0.283, meaning that both fixed and random effects explained 28.3% of the observed variance. However, the marginal *R* ^2^ was only 0.084, suggesting that the fixed effects alone accounted for only 8.4% of the observed variance. As in the vigilance model, this suggests that herd identity captured a substantial amount of the observed variation.

**FIGURE 4:**
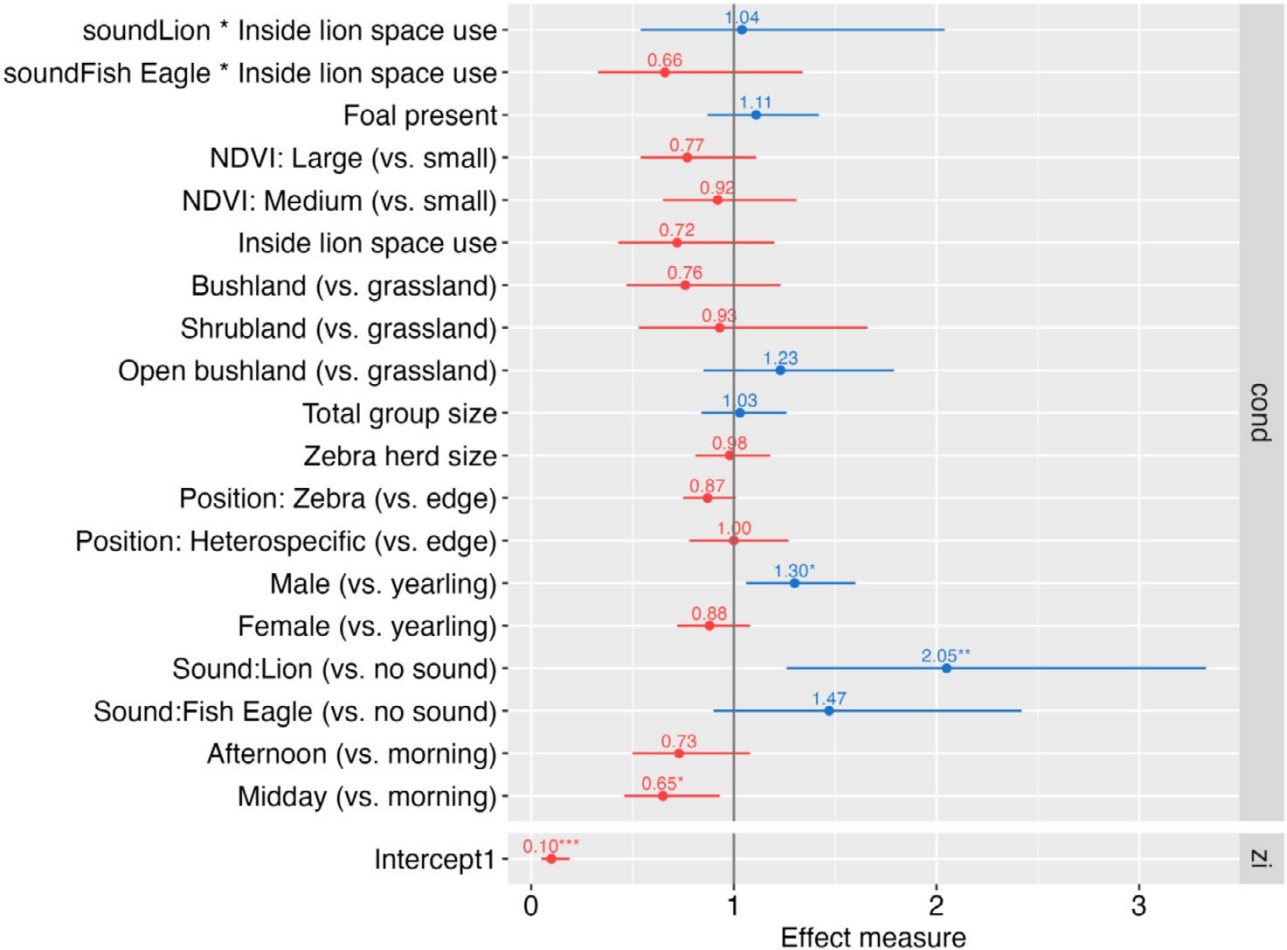
Effect measures of a generalized linear mixed model with negative binomial error distribution. The model consists of a zero‐inflation part (zi: Effect measure = odds ratio) which accounts for excess zeros, and a conditional part (cond: Effect measure: Incidence rate ratio) which models the counts of vigilance scans. The zero‐inflation sub‐model was best expressed by an intercept‐only specification. The conditional (count) sub‐model assesses the impact of environmental and zebra‐related variables and the impact of the playback sound (including the interaction between playback sound and the lion home range variable) on the proportion of vigilant scans of zebra. The proportion of vigilant scans is based on observations of zebras subject to playback experiments in Manyara Ranch, northern Tanzania.

Among the fixed effects, we found that the playback treatment mediated zebra behavior: When exposed to lion roar playbacks, zebras had higher vigilance levels compared to the no‐sound treatment (exp(β) = 2.05; 95% CI: 1.26–3.33, *p* = 0.004; Figure 4 for effect measures and Appendix B, Table B2). In addition, male zebras exhibited a significantly higher proportion of vigilant scans compared to yearlings (exp(β) = 1.30; 95% CI: 1.06–1.60, *p* = 0.011), and zebras were less vigilant during midday than in the morning (exp(β) = 0.65; 95% CI: 0.46–0.93, *p* = 0.019). The zero‐inflation part of the model was best explained by an intercept‐only term to differentiate between zero and ≥ 1 vigilance. Other variables, including the location of the zebra relative to the LSU, habitat type, NDVI class, total group size, zebra herd size, position within a group, foal presence, and the interaction between the playback sound and the LSU, did not significantly alter vigilance levels.

### 3.3 Flight Initiation Distance

The model explaining variation in FID fit the data well and appropriately captured the distribution of the target variable. The linearity assumption was approximately met, the variance of residuals was relatively homogeneous, collinearity was minimal, and the residuals followed a normal distribution (See Appendix C, Figure C3). However, the model explained only 12.4% of the observed variance (*R* ^2^ = 0.124).

**FIGURE 5:**
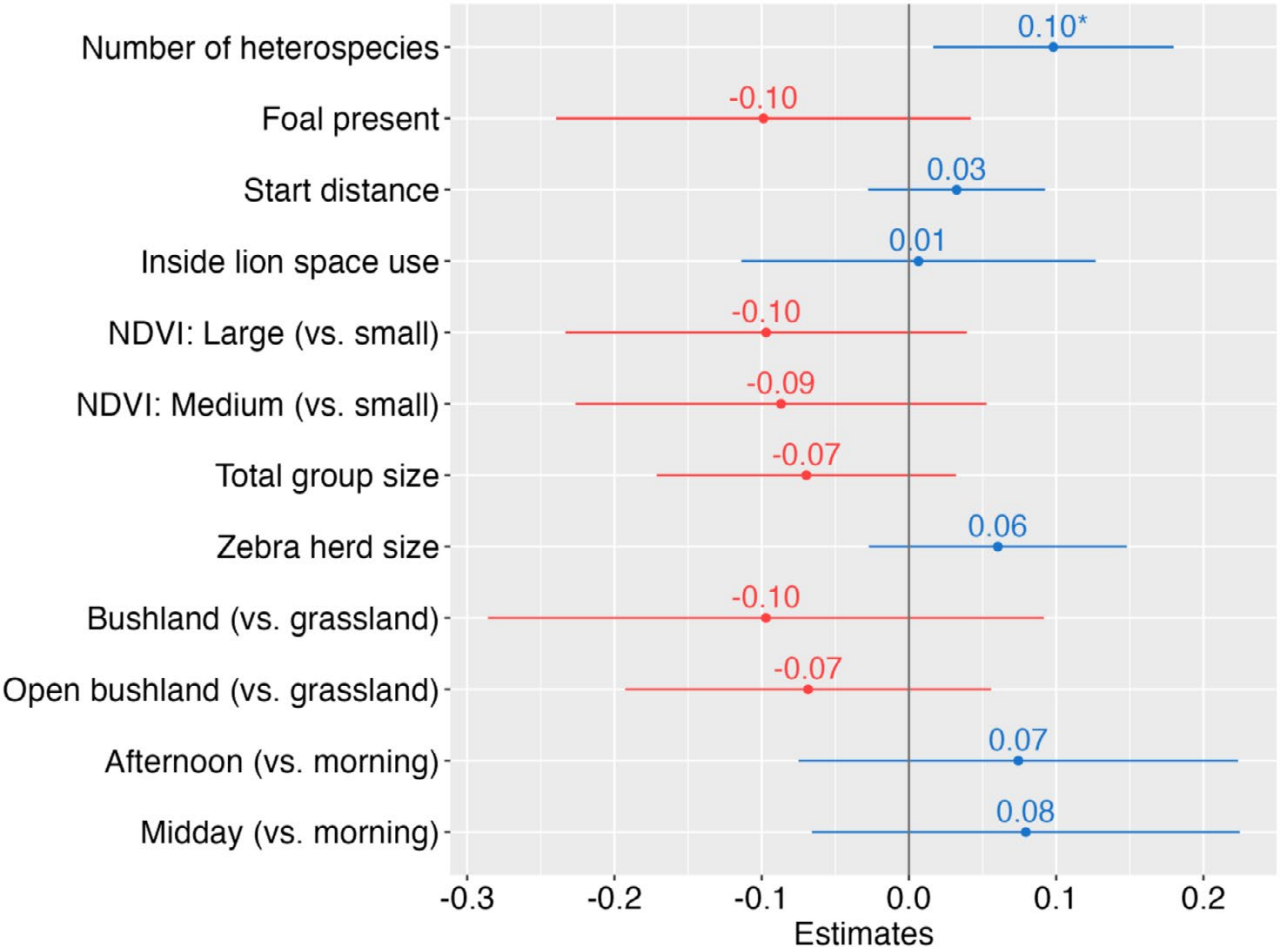
Regression coefficient estimates of a generalized linear model with Gaussian error distribution, testing the impact of environmental and zebra‐related variables on flight initiation distances of zebra in Manyara Ranch, northern Tanzania.

Only the number of heterospecifics was positively correlated with FID (β = 0.10; 95% CI: 0.02–0.18, *p* = 0.019; Figure 5, Appendix B, Table B3). Other considered variables, including location relative to LSU, habitat type, NDVI class, total group size, zebra herd size, and foal presence, did not significantly explain the observed variation in FID.

## 4 Discussion

Using three lines of experimental and observational assays, we did not find strong evidence that zebra risk perception was mediated by their location relative to lion space use—a key predator for zebra in our study area. While zebras increased vigilance in response to lion roars, this effect was independent of whether zebras were inside the LSU. Likewise, zebras inside the LSU did not adjust their FID when approached by a human, suggesting that fear of a predator is not generalizable to anthropogenic threats. Other spatial features such as habitat type and NDVI were also not associated with vigilance or FID. Instead, behavioral responses were influenced by grouping factors, with zebras in larger herds and those surrounded by conspecifics and heterospecifics being less vigilant. Zebras also fled at greater distances when associated with more heterospecific species. While our study revealed significant patterns, the models explained only a moderate portion of the variance in zebra vigilance and flight responses. Given the inherent complexity of field‐based behavioral research, where ecological, environmental, and social factors interact, we discuss potential methodological limitations and interpret our key results in a broader ecological context.

### 4.1 Methodological Considerations for Quantifying Predation Risk, Anthropogenic Influence, and Vigilance

One limitation of our study is that we worked with a small dataset of lions (*n* = 23 data points, possibly from multiple prides over 5 years) and used the MCP method to estimate LSU. We then adopted a binary measure of risk (i.e., inside or outside LSU). Creel et al. (2014) demonstrated that vigilance in five African ungulate species was better explained by the continuous distance between the ungulate herd and predators than by a binary presence/absence measure of predators. Therefore, it is quite possible that zebras adjust their risk perception, but this adjustment operates at a finer spatial grain. Hence, we recommend that future studies adopt a multi‐scale approach to describe predation risk (Moll et al. 2017). This could, for example, estimate lion space‐use intensity based on movement data collected at higher temporal resolution and exploring finer‐scale, quantified risk levels (see e.g., Middleton et al. 2013 and associated critique by Creel et al. 2013).

To quantify zebra risk perception, we used head‐up posture as a proxy for vigilance in two of our assays. Possibly, other forms of vigilance (i.e., when zebras did not obviously raise their heads) may have gone unnoticed. Maybe more importantly, we did not differentiate between types of vigilance, such as routine, social, and intense vigilance (Périquet et al. 2012). As social vigilance plays a key role in many species including zebras (Barnier et al. 2016), with individuals monitoring conspecifics for social positioning, competition, or access to mates, some portion of what we measured as “vigilance” functions to monitor group members rather than detect predators. This social vigilance is not only crucial in multiple monkey species (e.g., Hirsch 2002; Bernardi‐Gómez et al. 2023) but also in ungulates such as giraffes (Cameron and du Toit 2005). In our study, male zebras were more vigilant, while yearlings and females showed similar levels of vigilance. This pattern is consistent with previous research suggesting that plains zebra males are more vigilant than females possibly due to the need for monitoring rivals (Barnier et al. 2016). Similarly, non‐territorial male Przewalski's gazelles ( *Procapra przewalskii* ) were more vigilant toward other aggressive males during rutting season (Li et al. 2012). Given the likely overlap between social and antipredator vigilance, failing to distinguish between vigilance types may have obscured patterns related to risk perception. Accurately and consistently defining vigilance remains an issue in behavioral research (Allan and Hill 2018). Future studies could adopt a multi‐hypothesis framework to assess the relative contribution of different vigilance types under varying social and ecological conditions (e.g., distance from nearest male, number of males in a herd, rutting vs. non‐rutting season) (Allan and Hill 2018; Creel et al. 2014). Employing a reaction norm approach, where behavioral determinants can vary by individual, would provide additional insights into how vigilance is mediated. Additionally, continuous focal sampling could provide a more detailed assessment of vigilance duration, timing, and intensity in dynamic group settings (Allan and Hill 2018).

Finally, a potential human shield effect, where prey species perceive reduced predation risk in proximity to humans, could influence vigilance and flight responses (Goldenberg et al. 2017). While zebras face human threats through illegal exploitation (Kiffner et al. 2015) and exhibit heightened responsiveness to human observers (Kiffner et al. 2014), the extent to which human presence mediates zebra risk perception remains uncertain. Testing this hypothesis would require comparing behavioral assays conducted from concealed locations with those from more visible platforms, as in our study.

### 4.2 Response to Playback

In the playback experiments, zebras exhibited higher vigilance in the morning and afternoon but markedly lower vigilance at midday. This could reflect the timing of lion activity, which typically peaks in the morning, evening, and at night (note that we did not collect data at night) (Hayward and Hayward 2007). While we did not explicitly test for an interaction between time of day and playback treatment and the fixed effects of our models explained only a moderate amount of the observed variance, our findings suggest that zebra responses to immediate predation cues (i.e., lion roars) occur within a broader temporal landscape of risk. This aligns with Dröge et al. (2017) who showed that the behavior of multiple African ungulate species is also shaped by the temporal distribution of predation risk.

However, environmental and methodological factors may have also influenced our results. While we conducted playback experiments under comparable weather conditions (no experiments were conducted during rain or strong winds), finer‐scale factors such as sound propagation affected by wind or vegetation could have played a role (Fischer et al. 2013). Additionally, one could question the utility of using sound playbacks to simulate predator presence. In line with other researchers (Zanette et al. 2023), we used lion roars to simulate immediate lion presence, although lions typically do not roar when hunting. To address this potential mismatch, behavioral experiments with alternative modes for simulating immediate lion presence, such as full‐sized predator models or cutouts (Stankowich and Coss 2006), may provide more nuanced insights into zebra risk assessment. Despite these limitations, the strong increase in vigilance observed in response to lion playbacks (Figure 3) suggests that zebras adjust their risk perception in response to immediate predation risk (Courbin et al. 2016).

### 4.3 Flight Initiation Distance

Our FID experiment does not lend support to the hypothesis that fear is generalizable, as we did not detect differences in zebra flight responses relative to the broad‐scale distribution of lions. A potential explanation for the lack of spatial variation in FID is that different predator types elicit distinct antipredator strategies. Lions are primarily ambush predators who primarily sit and wait for their prey and then launch a surprise attack (Hopcraft et al. 2005), whereas humans in our study area primarily use pursuit tactics, such as chasing animals with motorbikes (Kiffner et al. 2014). Since zebras can likely differentiate between these distinct threats, their risk perception may be shaped more by immediate cues or learned experiences than by broad‐scale differences in lion distribution.

Another factor that may have contributed to the limited explanatory power of the FID model is unquantified individual differences in prior experience with humans. While zebras in our study spend substantial time outside protected areas (Kiffner et al. 2020b; Riggio et al. 2022), the frequency and severity of encounters with harmful human activities may differ across zebra herds. Accounting for individual identity in future studies, for example, using photo‐identification (Bolger et al. 2012), could provide additional insights, particularly regarding reaction norms and individual variation in FID. There is increasing recognition that individual animals differ in their responses to risk (Blumstein et al. 2023), and future research could explicitly model both individual‐level variation and reaction norms, offering a more nuanced understanding of how zebras (or other prey species) adjust their perception to varying risk factors.

Beyond individual variation, another key consideration is how zebras perceive different anthropogenic threats. Our FID experiment aimed to capture human‐associated risk using a single researcher (white, female, consistently dressed) who approached zebra herds in a standardized manner. Other species, such as Poeppigi's wooly monkeys ( *Lagothrix poeppigii* ) (Papworth et al. 2013) and elephants ( *Loxodonta africana* ) (McComb et al. 2014), can distinguish between different human behaviors and groups of people. If zebras similarly discriminate among human activities and traits, standardized FID experiments using researchers may not fully capture their risk perception of anthropocentric threats. Future studies could explore this by incorporating different human stimuli, such as motorbikes (which are often used by illegal hunters in this area) or pastoralists (Kiffner et al. 2014).

### 4.4 Is Zebra Risk Perception Constrained by Uncertainty?

While we cannot entirely reject the hypothesis that zebra risk perception is unaffected by broad‐scale variation in lion distribution, we offer several hypotheses that may help explain why we did not observe differences in vigilance and FID relative to LSU.

One possibility is informational constraints. The ecology of fear framework (Brown et al. 1999) assumes that prey have the knowledge about their spatial and temporal conditions and would modify their behavior accordingly. However, it is possible to consider that zebras may not have sufficient information to know that they are inside the LSU or areas associated with higher risks. Such ambiguity about predation risk is common (Dall et al. 2005; Munoz and Blumstein 2012; Feyten and Brown 2018). A number of studies suggest that when animals were uncertain about risk, they should overestimate risk (Bouskila and Blumstein 1992; Brown and Godin 2023; Brown et al. 2014). Error management theory (Johnson et al. 2013) suggests that under uncertainty, decisions are biased toward making less costly errors. For zebras, this could imply that rather than allocating antipredator behavior as a function of whether they are in risky places, they do not vary their assessments of risk because virtually all zebras are exposed to some risk of lion predation throughout their lives regardless of spatial variation in risk factors.

A possible mechanism explaining informational constraints could be that zebras have fundamental cognitive limitations on identifying risky places. We consider this unlikely because ungulates are known to have similar cortical mass and number of neurons as their carnivorous predators, and this conclusion might suggest that predators and prey went through analogous evolutionary trajectory and were able to cognitively navigate predatory interactions (Jardim‐Messeder et al. 2017). In addition, several empirical studies suggest that ungulates respond to spatial variation in predation risk (Schmidt 2019). For instance, red deer ( *Cervus elaphus* ) in Poland became more alert in the core of the wolves' ( *Canis lupus* ) territory than near the peripheral of the territory and also more vigilant when predator detection is constrained by vegetation (Kuijper et al. 2015). Similarly, wildebeest ( *Connochaetes taurinus* ) displayed higher vigilance in places with greater long‐term risk (Dröge et al. 2017). Therefore, we doubt that zebras in our study were fundamentally incapable of estimating spatial variation in predation risk.

Alternatively, zebras may not have been constrained by informational constraints. Again, regardless of whether or not they were in a risky place, all zebras faced some risk of predation by lions. In our study area, lions are the primary predator of zebra during the daytime, and during their lifetime, zebras must avoid lion predation, regardless of where they are. In addition, lion home ranges and hunting forays vary (Loveridge et al. 2009) and may be generally unpredictable due to the possibility of dispersing lions. Furthermore, zebras may also face predation threats from other species. In our study area, additional mortality risk may primarily arise from spotted hyena predation (though these are primarily nocturnal) and illegal hunting by humans. Thus, the background risk might be relatively uniform to zebras across the entire study area. If the risk is uniform and sufficiently high, we would not expect spatial variation in LSU to influence zebra's perceptions of risk.

### 4.5 Grouping as an Alternative Strategy to Mitigate Predation Risk

Finally, zebras could be aware of the lions' whereabouts and other spatial risk features, but rather than relying on spatial clues alone, zebras mitigate predation risk by alternative behaviors. Grouping provides multiple antipredator benefits, including diluting individual mortality risk (Foster and Treherne 1981) and increasing predator detection (Pulliam 1973). Previous studies suggested that detection plays a greater role in mixed‐species herds and that dilution depends on the extent to which the coexisting species are considered favorable prey to common predators (Schmitt et al. 2014).

Our results indicate that grouping explained some variation in our response variables, supporting findings from previous studies on ungulate behavior. Creel et al. (2014) found that vigilance levels of five ungulate species (zebra, wildebeest, Grant's gazelle *Nanger granti* , impala *Aepyceros melampus* , and giraffe) were negatively correlated with group size. Additionally, zebras associated with giraffes exhibited reduced vigilance compared to when in conspecific groups, likely relying on cues from giraffes to assess predation risk (Schmitt et al. 2016). In support of this, Kiffner et al. (2022) showed that the occurrence of mixed species groups was higher inside the LSU, with ungulate species more likely to associate with heterospecifics including giraffes. This pattern was largely driven by similarity in prey preferences among lions, suggesting that predation risk is a key driver for the composition of mixed species groups.

In our study, both larger group size and central positioning within the herd were associated with reduced vigilance, further emphasizing the role of grouping in modulating antipredator behavior. Future research could explore whether group geometry and individual spatial positioning within herds are more influential in certain risk contexts and whether these factors vary depending on associated species. As group geometry likely interacts with herd size, studying these dynamics could provide deeper insights into how zebras optimize their antipredator strategies in mixed‐ and single‐species groups.

## 5 Conclusions

Our study does not provide strong evidence that zebras adjust their vigilance or flight responses based on broad‐scale lion distribution. While we cannot fully reject this hypothesis, our results suggest that zebras primarily mitigate predation risk by relying on direct predator cues and adjusting grouping strategies. Moreover, zebra responses varied widely and were strongly associated with herd identity, indicating intergroup‐level plasticity or the influence of unmeasured context‐dependent factors. Future research would benefit from a more comprehensive spatiotemporal approach, particularly incorporating nocturnal risk assessment when lions are most active. Additionally, using a reaction‐norm framework could help disentangle behavioral plasticity from individual variation.

## Author Contributions

**Yuchen Chen:** conceptualization (equal), data curation (equal), formal analysis (lead), investigation (equal), methodology (equal), validation (equal), visualization (lead), writing – original draft (lead). **Daniel T. Blumstein:** conceptualization (equal), formal analysis (equal), methodology (equal), project administration (equal), supervision (lead), validation (equal), writing – review and editing (lead). **Diana M. Boyle:** conceptualization (equal), data curation (lead), funding acquisition (equal), investigation (lead), methodology (equal), validation (equal), writing – review and editing (equal). **Natasha Bartolotta:** data curation (equal), investigation (equal), validation (equal), writing – review and editing (supporting). **Jessica Brown:** data curation (equal), investigation (equal), validation (equal), writing – review and editing (supporting). **Bernard Kissui:** data curation (equal), investigation (equal), visualization (supporting), writing – review and editing (supporting). **Matthias Waltert:** project administration (supporting), supervision (equal), validation (supporting), writing – review and editing (supporting). **Christian Kiffner:** conceptualization (lead), data curation (equal), formal analysis (equal), funding acquisition (supporting), investigation (equal), methodology (lead), project administration (lead), supervision (equal), validation (equal), visualization (equal), writing – review and editing (lead).

## Conflicts of Interest

The authors declare no conflicts of interest.

## Supporting information


Appendix S1.

## Data Availability

Raw data and accompanying code are available at GRO.data: https://doi.org/10.25625/FB9FOA.
