# Supplementary material for: Zebra Risk Perception in a Landscape of Fear
Source: Ecol Evol. 2025 May 14;15(5):e71275. doi: 10.1002/ece3.71275 (PMC12077961; doi:10.1002/ece3.71275)

**Appendix A** **Model selection tables**

Table A1 AICc scores of negative binomial models, testing the impact of environmental and zebra-related variables on the proportion of vigilant scans of zebra from behavioral observation in Manyara Ranch, northern Tanzania.

| Negative Binomial Type | Zero-inflation and predictor | AICc |
| --- | --- | --- |
| nbinom1 | No Zero-inflation | 4180.0 |
| nbinom1 | Zero-inflation simple (without a predictor) | 4165.7 |
| nbinom1 | Zero-inflation with lion space use as a predictor | 4165.1 |
| nbinom1 | Zero-inflation with zebra’s position in a herd as a predictor | 4156.8 |
| nbinom2 | No Zero-inflation | 4234.8 |
| nbinom2 | Zero-inflation simple (without a predictor) | 4232.9 |
| nbinom2 | Zero-inflation with lion space use as a predictor | 4231.3 |
| nbinom2 | Zero-inflation with zebra’s position within a herd as a predictor | 4214.0 |

Table A2 AICc scores of negative binomial models, testing the impact of environmental and zebra-related variables on the proportion of vigilant scans of zebra subject to playback experiments in Manyara Ranch, northern Tanzania.

| Negative Binomial Type | Zero-inflation and predictor | AICc |
| --- | --- | --- |
| nbinom1 | No Zero-inflation | 2935.1 |
| nbinom1 | Zero-inflation simple (without a predictor) | 2915.3 |
| nbinom1 | Zero-inflation with lion space use as a predictor | 2917.5 |
| nbinom1 | Zero-inflation with zebra’s position within a herd as a predictor | 2919.3 |
| nbinom2 | No Zero-inflation | 3016.7 |
| nbinom2 | Zero-inflation simple (without a predictor) | 2962.2 |
| nbinom2 | Zero-inflation with lion space use as a predictor | Model did not converge |
| nbinom2 | Zero-inflation with zebra’s position within a herd as a predictor | 2961.3 |

**Appendix B Model results summary**

Table B1 Exponentiated regression coefficient estimates of a generalized linear mixed model with negative binomial error distribution. The model consists of a zero-inflation part (zi) which accounts for excess zeros, and a conditional part which models the counts of vigilance scans. For the zero-inflation sub-model, we included the zebra’s position within the herd as a predictor. The conditional (count) sub-model assesses the impact of multiple environmental and zebra-related variables on the proportion of vigilant scans of zebra. The conditional (count) sub-model assesses the impact of multiple environmental and zebra-related variables on the proportion of vigilant scans of zebra. The proportions of vigilant scans are based on behavioral observations in Manyara Ranch, northern Tanzania.

| **Characteristic** | **exp(Beta)** | **95% CI***^1^* | **p-value** |
| --- | --- | --- | --- |
| **Conditional:** | | | |
| **Time of the day** |  |  |  |
| *Midday (vs. morning)* | 1.27 | 0.82, 1.95 | 0.278 |
| *Afternoon (vs. morning)* | 0.97 | 0.59, 1.59 | 0.902 |
| *Evening (vs. morning)* | 1.04 | 0.59, 1.83 | 0.903 |
| **Demographic** |  |  |  |
| *Female (vs. yearling)* | 0.89 | 0.70, 1.12 | 0.322 |
| *Male (vs. yearling)* | 1.78 | 1.40, 2.25 | **<0.001** |
| **Position** |  |  |  |
| *Heterospecific (vs. edge)^2^* | 0.54 | 0.41, 0.72 | **<0.001** |
| *Zebra (vs. edge)^3^* | 0.86 | 0.70, 1.07 | 0.172 |
| **Zebra herd size** | 1.24 | 0.97, 1.58 | 0.085 |
| **Total group size** | 0.78 | 0.61, 1.00 | **0.047** |
| **Habitat** |  |  |  |
| *Open bushland (vs. grassland)* | 1.22 | 0.84, 1.76 | 0.298 |
| *Bushland (vs. grassland)* | 1.10 | 0.70, 1.73 | 0.679 |
| **NDVI** |  |  |  |
| *Medium (vs. small)* | 1.22 | 0.82, 1.82 | 0.322 |
| *Large (vs. small)* | 1.20 | 0.80, 1.80 | 0.383 |
| **Lion space use** |  |  |  |
| *TRUE* | 0.95 | 0.64, 1.41 | 0.811 |
| **Foal** |  |  |  |
| *TRUE* | 1.27 | 0.96, 1.69 | 0.099 |
| **Zero-Inflation:** | | | |
| **Position** |  |  |  |
| *Heterospecific (vs. edge)^2^* | 0.00 | 0.00, Inf | 0.996 |
| *Zebra (vs. edge)^3^* | 3.85 | 1.98, 7.49 | **<0.001** |
| *^1^*CI = Confidence Interval | | | |
| *^2^*In a heterospecific group (vs. at the edge of a zebra group) | | | |
| *^3^*Inside a zebra group (vs. at the edge of a zebra group) | | | |

Table B2 Exponentiated regression coefficient estimates of a zero-inflated negative binomial model with conditional part and zero-inflation part without a predictor, testing the impact of environmental and zebra-related variables on the proportion of vigilant scans of zebra exposed to playback experiments in Manyara Ranch, northern Tanzania.

| **Characteristic** | **exp(Beta)** | **95% CI***^1^* | **p-value** |
| --- | --- | --- | --- |
| **Time of the day** |  |  |  |
| *Midday (vs. morning)* | 0.65 | 0.46, 0.93 | **0.019** |
| *Afternoon (vs. morning)* | 0.73 | 0.50, 1.08 | 0.121 |
| **Sound** |  |  |  |
| *Fish eagle (vs. no sound)* | 1.47 | 0.90, 2.42 | 0.124 |
| *Lion (vs. no sound)* | 2.05 | 1.26, 3.33 | **0.004** |
| **Demographic** |  |  |  |
| *Female (vs. yearling)* | 0.88 | 0.72, 1.08 | 0.219 |
| *Male (vs. yearling)* | 1.30 | 1.06, 1.60 | **0.011** |
| **Position** |  |  |  |
| *Heterospecific (vs. edge)^2^* | 1.00 | 0.78, 1.27 | 0.980 |
| *Zebra (vs. edge)^3^* | 0.87 | 0.75, 1.01 | 0.076 |
| **Zebra herd size** | 0.98 | 0.81, 1.18 | 0.799 |
| **Total group size** | 1.03 | 0.84, 1.26 | 0.756 |
| **Habitat** |  |  |  |
| *Open bushland (vs. grassland)* | 1.23 | 0.85, 1.79 | 0.269 |
| *Shrubland (vs. grassland)* | 0.93 | 0.53, 1.66 | 0.815 |
| *Bushland (vs. grassland)* | 0.76 | 0.47, 1.23 | 0.265 |
| **Lion space use** |  |  |  |
| *TRUE* | 0.72 | 0.43, 1.20 | 0.207 |
| **NDVI** |  |  |  |
| *Medium (vs. small)* | 0.92 | 0.65, 1.31 | 0.645 |
| *Large (vs. small)* | 0.77 | 0.54, 1.11 | 0.157 |
| **Foal** |  |  |  |
| *TRUE* | 1.11 | 0.87, 1.42 | 0.388 |
| **Sound * Lion space use** |  |  |  |
| *soundFish eagle * TRUE* | 0.66 | 0.33, 1.34 | 0.252 |
| *soundLion * TRUE* | 1.04 | 0.54, 2.04 | 0.900 |
| *^1^*CI = Confidence Interval | | | |
| *^2^*In a heterospecific group (vs. at the edge of a zebra group) | | | |
| *^3^*Inside a zebra group (vs. at the edge of a zebra group) | | | |

Table B3 Regression coefficient estimates of a generalized linear model with Gaussian error distribution, testing the impact of environmental and zebra-related variables on flight initiation distances of zebra in Manyara Ranch, northern Tanzania.

| **Characteristic** | **Beta** | **95% CI***^1^* | **p-value** |
| --- | --- | --- | --- |
| **Time of the day** |  |  |  |
| *Midday (vs. morning)* | 0.08 | -0.07, 0.22 | 0.284 |
| *Afternoon (vs. morning)* | 0.07 | -0.07, 0.22 | 0.329 |
| **Habitat** |  |  |  |
| *Open bushland (vs. grassland)* | -0.07 | -0.19, 0.06 | 0.280 |
| *Bushland (vs. grassland)* | -0.10 | -0.29, 0.09 | 0.313 |
| **Zebra herd size** | 0.06 | -0.03, 0.15 | 0.177 |
| **Total group size** | -0.07 | -0.17, 0.03 | 0.179 |
| **NDVI** |  |  |  |
| *Medium (vs. small)* | -0.09 | -0.23, 0.05 | 0.223 |
| *Large (vs. small)* | -0.10 | -0.23, 0.04 | 0.163 |
| **Lion space use** |  |  |  |
| *TRUE* | 0.01 | -0.11, 0.13 | 0.916 |
| **Start distance** | 0.03 | -0.03, 0.09 | 0.291 |
| **Foal** |  |  |  |
| *TRUE* | -0.10 | -0.24, 0.04 | 0.169 |
| **Number of heterospecifics** | 0.10 | 0.02, 0.18 | **0.019** |
| *^1^*CI = Confidence Interval | | | |

**Appendix C Model check**

Figure C1 Model fit indicators (posterior predictive check, overdispersion and zero-inflation, homogeneity of variance, collinearity, normality of residuals, and normality of random effects) for a generalized linear mixed model with negative binomial error distribution and accounting for zero-inflation, describing the proportion of vigilant scans of zebras in Manyara Ranch, northern Tanzania. The posterior predictive check indicates that the model occasionally predicts vigilance counts exceeding the theoretical maximum of 13, highlighting a limitation in capturing the capped nature of the response variable.


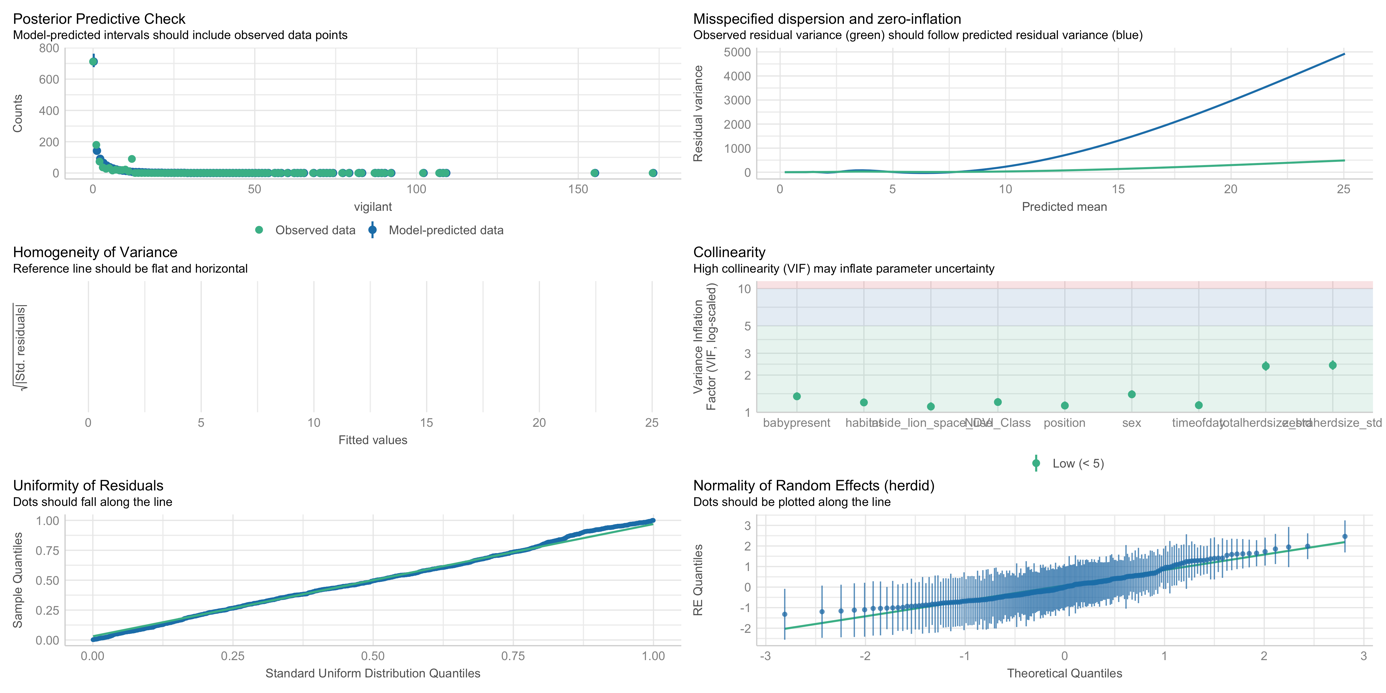


Figure C2 Model fit indicators (posterior predictive check, overdispersion and zero-inflation, homogeneity of variance, collinearity, normality of residuals, and normality of random effects) for a generalized linear mixed model with negative binomial error distribution and accounting for zero-inflation, describing the proportion of vigilant scans of zebras subject to playback experiments in Manyara Ranch, northern Tanzania. The posterior predictive check indicates that the model occasionally predicts vigilance counts exceeding the theoretical maximum of 12, highlighting a limitation in capturing the capped nature of the response variable.


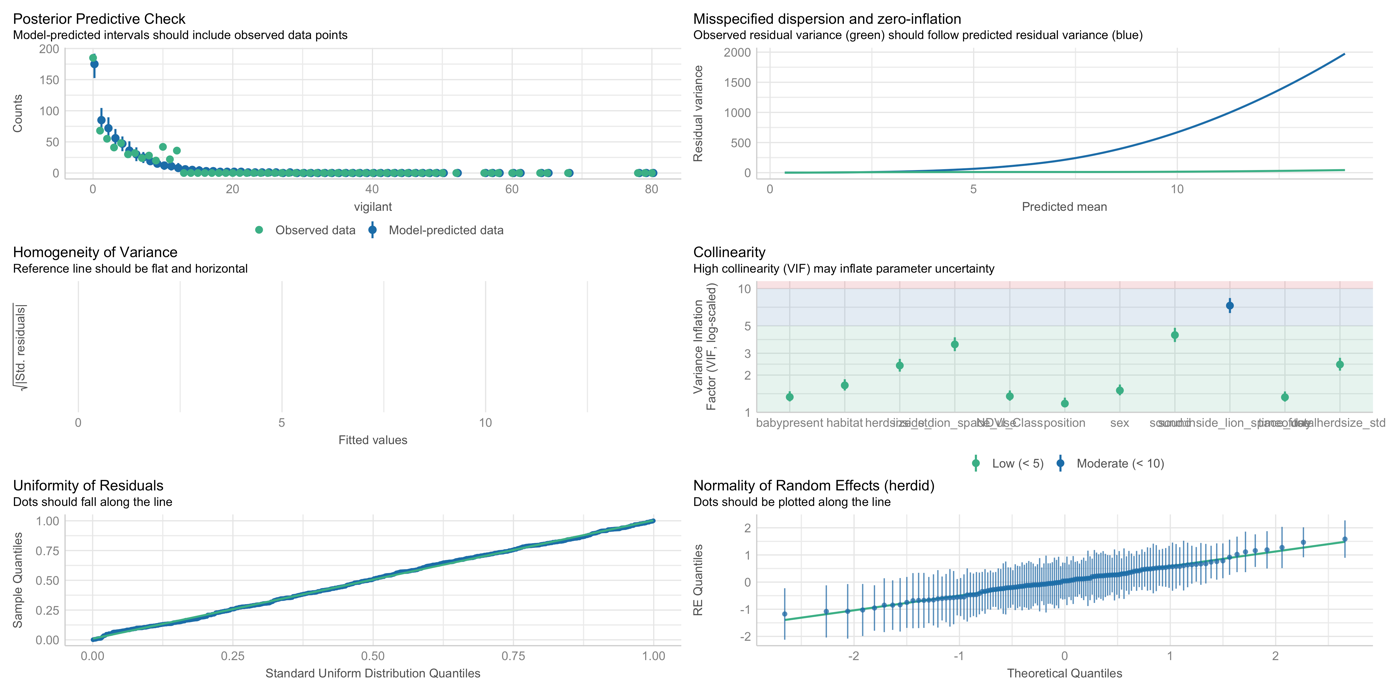


Figure C3 Model fit indicators (posterior predictive check, linearity, homogeneity of variance, collinearity, and normality of residuals) for a general linear model with Gaussian error distribution, to describe variation in flight initiation distances of zebras in Manyara Ranch, Tanzania.


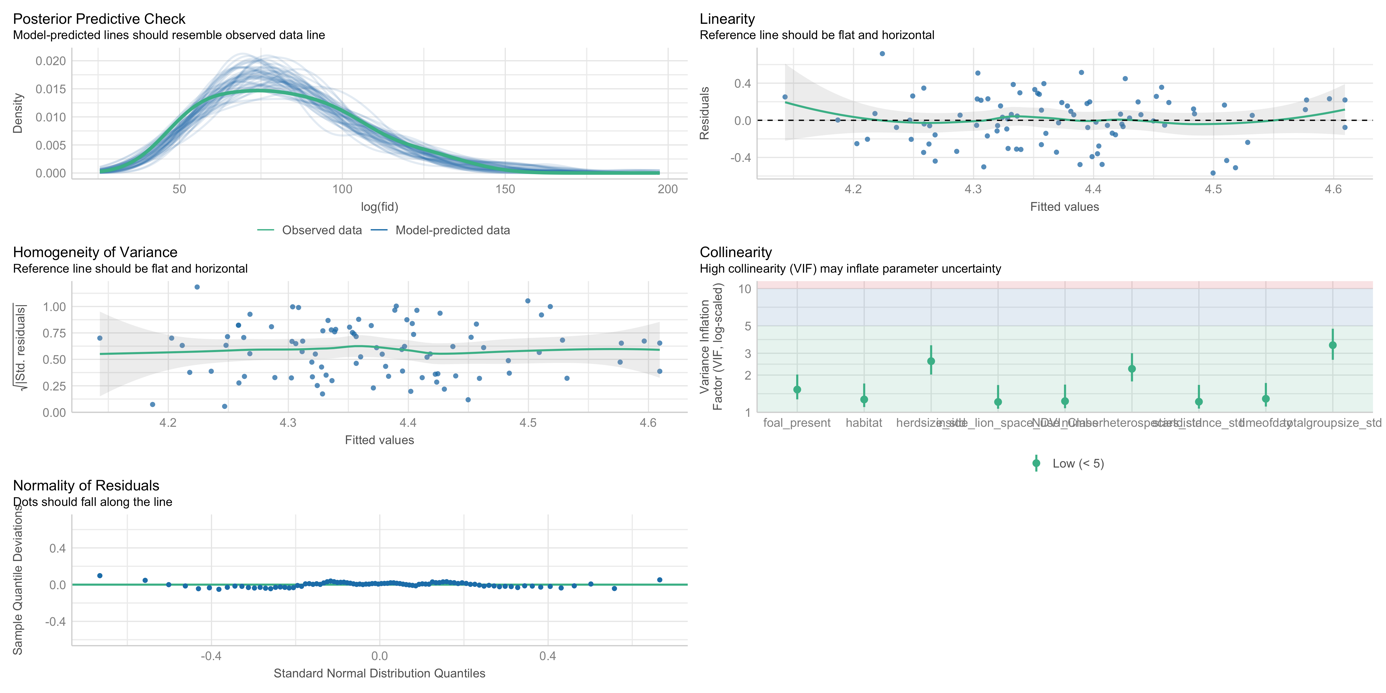

Supplement: Supplementary file 1 — Appendix S1. [file ECE3-15-e71275-s001.docx]
